# Hospital Capacity Data and Extreme Heat Event Vulnerability

**DOI:** 10.1001/jamanetworkopen.2024.32578

**Published:** 2024-09-11

**Authors:** Hussam Mahmoud, Meghana Gadgil, Emad M. Hassan, Michael Martin, Enrico G. Castillo, Stephanie Diem, Elena Krieger

**Affiliations:** 1 New Voices, Cohort 2, National Academy of Sciences, Engineering, and Medicine, Washington, DC; 2 Department of Civil and Environmental Engineering, Colorado State University, Fort Collins; 3 One Health Institute, Colorado State University, Fort Collins; 4 Division of Hospital Medicine, University of California, San Francisco; 5 Division of Health Policy and Management, School of Public Health, UC Berkeley, Berkeley, California; 6 Department of Psychiatry & Biobehavioral Sciences, University of California, Los Angeles; 7 Engineering Physics Department, the University of Wisconsin-Madison, Madison; 8 PSE Healthy Energy, Oakland, California

## Abstract

This qualitative study examines how regional health care capacity is associated with extreme heat event vulnerability.

## Introduction

Climate change is associated with an increase in the intensity, frequency, and duration of extreme heat events (EHEs).^1^ Governmental organizations rely on a variety of heat vulnerability indices (HVIs) to characterize risk and inform adaptation and mitigation policies. However, most HVIs fail to include metrics of health care capacity, and none have been included at a regional scale.^1^ Here, we investigate how incorporating regional health care capacity changes our understanding of EHE vulnerability.

## Methods

For this decision analytical modeling study, we developed a metric, hospital capacity in extreme heat (HCEH), by combining 3 normalized indices: summertime extreme heat days, population older than 65 years, and county’s number of hospital beds per capita. The number of heat days per county is based on a threshold represented by the 99th percentile of daily maximum temperature. We compare the HCEH with extreme heat vulnerability (EHV), calculated without including hospital beds per capita. We followed the CHEERS reporting guideline without cost analysis. We were exempt by our institutions from obtaining institutional review board approval and informed consent because this study does not constitute human participants research, in accordance with 45 CFR §46.

We used a moderate population growth projection^2^ and age older than 65 years as a proxy for individual heat vulnerability. Other at-risk groups, such as children, outdoor workers, and first responders, were excluded from the projections. We used a Representative Concentration Pathway (RCP 8.5), representing carbon concentration that causes global warming of 8.5 W/m^2^. The eAppendix in Supplement 1 provides additional information.^3,4,5^

## Results

**Figure 1.**
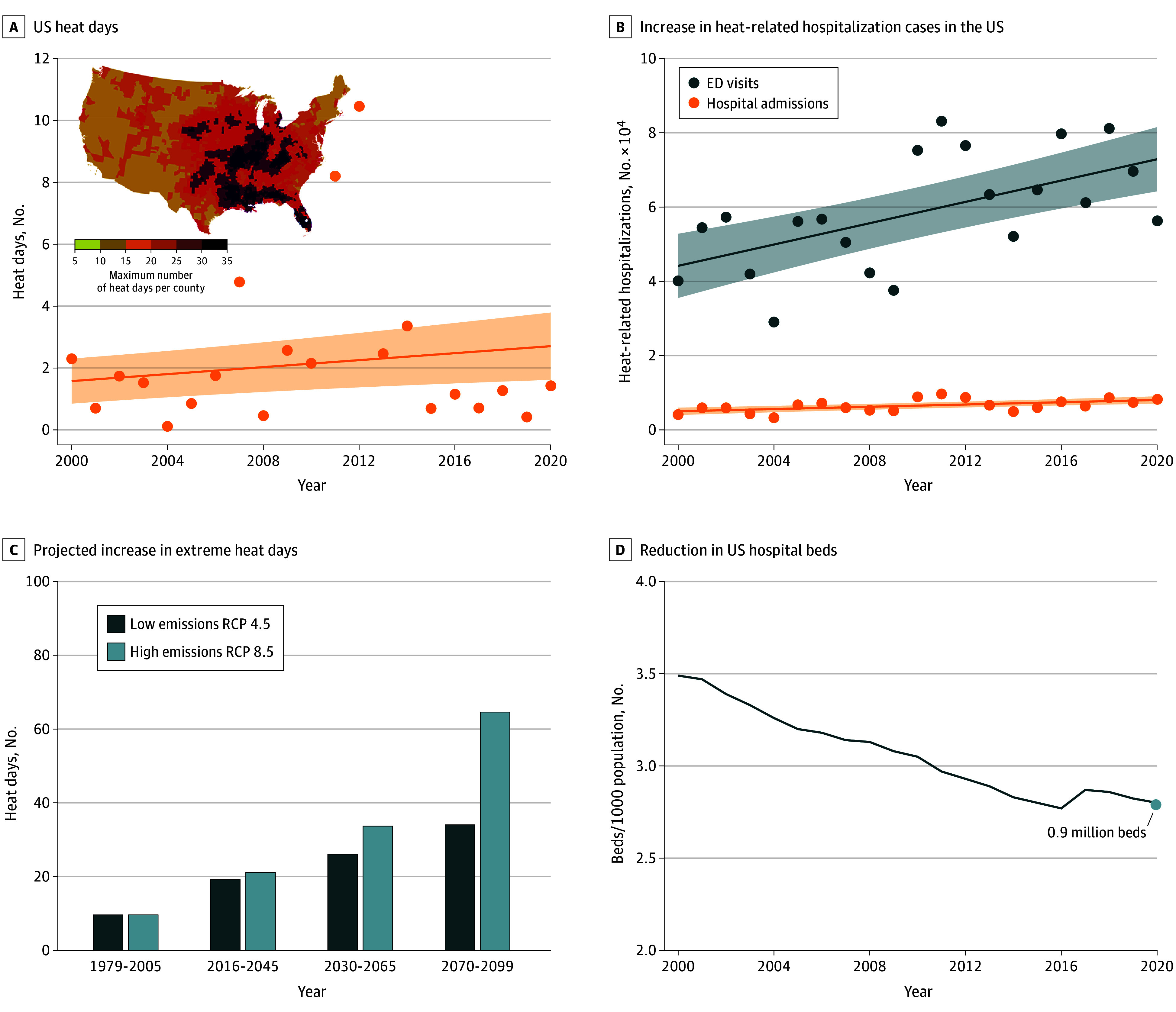
US Heat Days, Heat-Related Hospitalizations, and Expected Reductions in US Hospital Beds A, Graph shows the mean number of heat days calculated according to the data reported by the Centers for Disease Control and Prevention^3^ for US counties using relative threshold, 99th percentile-heat metric, and daily maximum temperature. The figure includes the change in the spatial distribution of the maximum number of heat waves per year between 1979 and 2020.^3^ Line denotes the mean, shaded areas denote the 95% CI, and dots denote outliers. B, Graph shows the increase in the total number of heat-related hospitalization cases in the US,^3^ including emergency department (ED) visits (increase of 1436 per year) and hospital admissions (increase of 153 per year). Lines denote the means, shaded areas denote the 95% CI, and dots denote outliers. C, Graph shows the projected increase of the number of extreme heat days for 2 different climate scenarios, Representative Concentration Pathway (RCP) 4.5 and RCP 8.5. D, Graph shows the reduction in the total number of US hospital beds.^5^

Figure 1A shows the yearly maximum number of extreme heat days and EHEs by county collected between 1979 and 2020, from the Centers for Disease Control and Prevention.^3^ On the basis of statistical analysis for data from the past 2 decades,^3^ we project that heat wave–related morbidity during the summer months is likely to increase over time, as reflected in more emergency department (ED) visits and hospital admissions (Figure 1B). Heat-related ED visits and hospitalizations have increased precipitously, concentrated in geographic areas of the US that are more vulnerable to heat.^4^

In recent decades, the US’s average number of extreme heat days increased more than 8-fold (Figure 1C) and is expected to increase by a factor of up to 2.5 and 5.7 for 2 different representative CO_2_ concentration pathways—RCP 4.5 and RCP 8.5, respectively—by 2099. Certain regions, such as the Southeastern US, face an elevated threat from escalating humidity as well.^4^

The US has also been losing hospital capacity over the past decades. Figure 1D illustrates this reduction in hospital beds,^5^ with less decline in recent years of approximately 2% between 2015 and 2020. A recent analysis demonstrates that 80% of US counties are health care deserts.^6^

**Figure 2.**
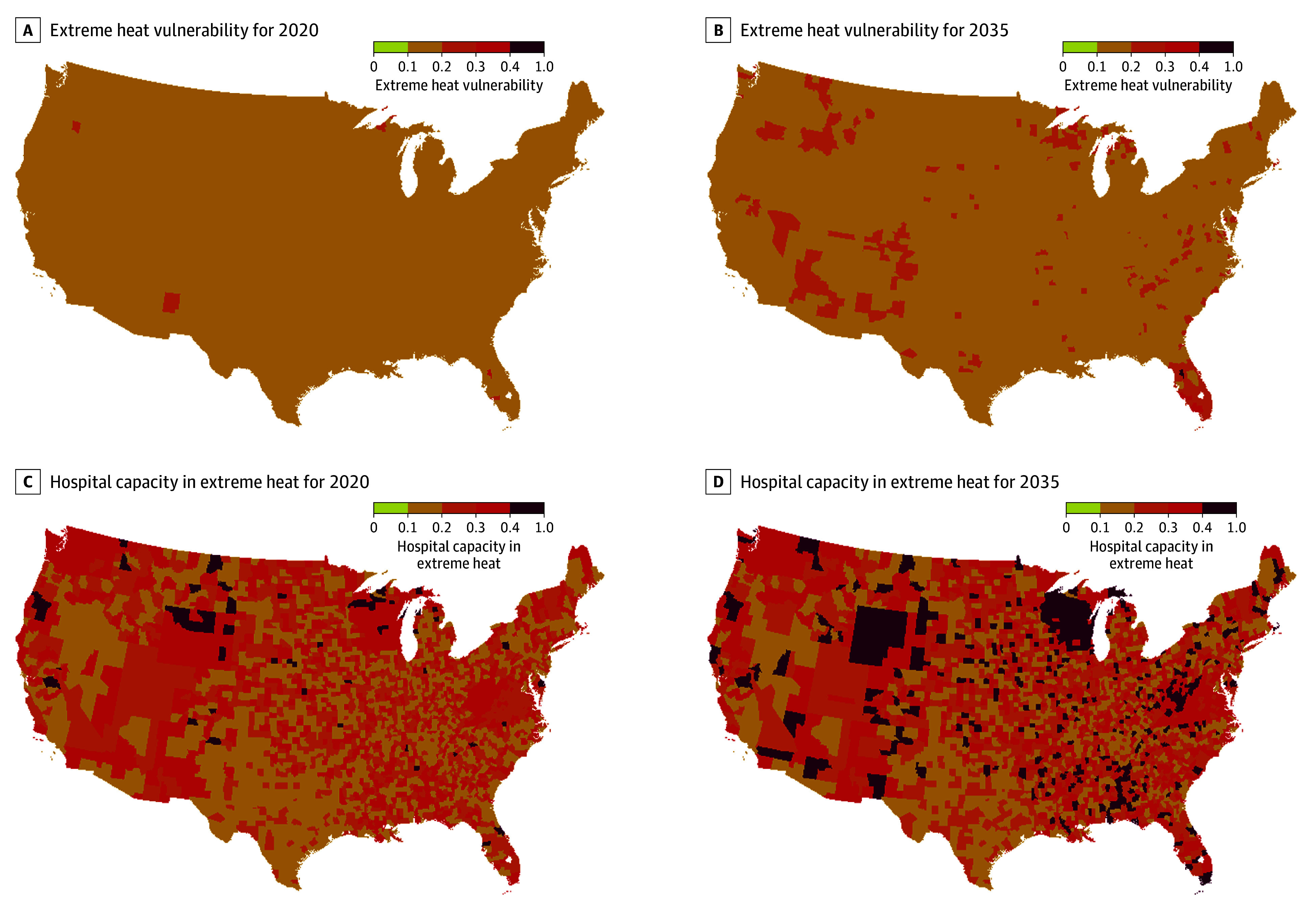
Extreme Heat Vulnerability and Hospital Capacity in Extreme Heat in the US Figure shows the extreme heat vulnerability for 2020 (A) and 2035 (B), and hospital capacity in extreme heat for 2020 (C) and 2035 (D).

Figure 2A and Figure 2B show the EHV, without hospital beds, for 2020 and 2035, respectively. Figure 2C and Figure 2D show the HCEH, including hospital beds, for 2020 and 2035, respectively. The higher the EHV and the HCEH indices, the worse the impact of heat waves. The figures show that HCEH is expected to increase greatly in the next decade, highlighting the urgency of applying different effective preventive measures.

## Discussion

A key finding from this decision analytical modeling study is the major increase in the magnitude and change in distribution of vulnerability to EHEs when we include health care system capacity. Both changes can be attributed to fewer hospital beds available to accommodate an increasing number of patients vulnerable to heat illness.

This study has some limitations. First, data on hospital beds per capita were readily available, but this metric overlooks key dimensions of true health system capacity like public health, primary care, and emergency services. Our model assumed a constant number of hospital beds per 100 000 people from 2020 onward, which may be optimistic given current trends. This model did not include the impact of EHE on workforce health, medical supplies, and supporting infrastructure. Our calculation of the HCEH extends to 2035 only, since estimates beyond 15 years from 2020 are likely to be highly uncertain.

## Notes

Supplement 1. **eAppendix. **Supplemental Methods
eReferences

Supplement 2.
Data Sharing Statement

## References

[1] US Global Change Research Program. Review of current comprehensive heat vulnerability and adaptation indices: USA regional differences and gaps in knowledge. September 2023. Accessed August 1, 2024. https://downloads.globalchange.gov/cchhg/Review-of-Current-Comprehensive-Heat-Vulnerability-and-Adaptation-Indices.pdf

[2] Hauer ME. Population projections for U.S. counties by age, sex, and race controlled to shared socioeconomic pathway. Sci Data. 2019;6:190005. doi:10.1038/sdata.2019.5 30720801 PMC6362894

[3] Centers for Disease Control and Prevention. National environmental public health tracking network. Data Explorer. 2020. Accessed August 1, 2024. https://ephtracking.cdc.gov/DataExplorer/

[4] Coffel ED, Horton RM, de Sherbinin A. Temperature and humidity based projections of a rapid rise in global heat stress exposure during the 21st century. Environ Res Lett. 2018;13(1):014001. doi:10.1088/1748-9326/aaa00e 32818039 PMC7430505

[5] World Bank. Hospital beds (per 1,000 people)—United States. 2020. Accessed August 1, 2024. https://data.worldbank.org/indicator/SH.MED.BEDS.ZS?locations=US

[6] Nguyen A, Kim S. Mapping healthcare deserts: 80% of the country lacks adequate access to healthcare. The GoodRx Research Team. September 9, 2021. Accessed August 1, 2024. https://www.goodrx.com/healthcare-access/research/healthcare-deserts-80-percent-of-country-lacks-adequate-healthcare-access

