# Supplementary material for: Hospital Capacity Data and Extreme Heat Event Vulnerability
Source: JAMA Netw Open. 2024 Sep 11;7(9):e2432578. doi: 10.1001/jamanetworkopen.2024.32578 (PMC11391326; doi:10.1001/jamanetworkopen.2024.32578)
Supplement: Supplement 1. — eAppendix. Supplemental Methods eReferences [file jamanetwopen-e2432578-s001.pdf]

Supplemental Online Content

Mahmoud H, Gadgil M, Hassan EM, et al. Hospital capacity data and extreme heat event vulnerability. *JAMA Netw Open*. 2024;7(9):e2432578.
doi:10.1001/jamanetworkopen.2024.32578

eAppendix. Supplemental Methods
eReferences

This supplemental material has been provided by the authors to give readers additional information about their work.

© 2024 Mahmoud H et al. *JAMA Network Open.*

eAppendix. Supplemental Methods

Methodological notes

The expected vulnerability of US counties to heat-related illness is quantified using the *HCEH* index. The $HCEH_i^t$, shown in Equation (1), represents the hospital capacity for county *i* subjected to extreme heat at any time *t*. The equation uses the number of summertime extreme heat days, *HD*, normalized by the total number of summer days (92 days). Additionally, the ratio between the population over age 65, P_{+65}, and the total population per county is used as a rough proxy for increased vulnerability to heat illness. Moreover, the number of hospital beds per capita, *B*, in each county, normalized by the maximum number of staffed beds per county, $\max B$, is utilized as the healthcare system capacity.

$$HCEH_i^t = \left(\frac{\sum HD_i^t}{92}\right)\left(\frac{\sum P_{+65i}^t}{\sum P_i^t}\right)\left(\frac{B_i}{\max\limits_i B_i}\right) \quad (1)$$

We used data provided by CDC (1) to determine the difference between the projected and historical heat days, *HDD*, in the summer months calculated based on the emission scenario RCP 8.5. The projected extreme heat days data are calculated based on a relative threshold considering a 99th percentile heat metric and daily maximum temperature. We calculated the projected number of heat days by adding the average number of historical heat days to the *HDD* collected from the CDC, as shown in the equation below.

$$HD_i^t = HDD_i^t + \frac{\sum_{1976}^{2005} HD_i^{\mathrm{t}}}{30} \quad (2)$$

We utilized Hauer's 'middle-of-the-road' population growth model (2). The model utilizes the cohort-component method to calculate the population per county, P^t, at any time *t*, as a function of the fertility, mortality, and migration variation, as shown in Equation (3). Where B^t, D^t, and $M^t_{in/out}$ are births, deaths, and in or out-migration at time *t*, respectively. The model uses the cohort-change ratio as an input for the Hamilton-Perry model to evaluate the population projections for different groups, including those over age 65, used in this study.

$$P_i^{t+1} = P_i^t + B_i^t - D_i^t + M_{in}^t - M_{out}^t \quad (3)$$

In addition, we conservatively assumed that the current (i.e., 2020) ratio of hospital beds per capita would be constant in the coming decades.

The Historical number of heat days is calculated based on the CDC (1) data for US counties. The heat-related hospital demand data is collected from the National Environmental Public Health Tracking Network (1). The historical data for the US number of beds per capita is collected from the World Bank (3). The projected heat days for the years 2035 are available on the National Environmental Public Health Tracking Network (1). The population growth data used to project the total heat-related demand for each county are collected for the years 2035 from (2).

© 2024 Mahmoud H et al. *JAMA Network Open.*

eReferences

1. Centers for Disease Control and Prevention, National environmental public health tracking network. Data Explorer https://ephtracking.cdc.gov/DataExplorer/ (2020).

2. M.E. Hauer, Population projections for U.S. counties by age, sex, and race controlled to shared socioeconomic pathway. *Sci. Data*, ***6***, 190005 (2019).

3. The World Bank, Hospital beds (per 1,000 people) - United States. Accessed [05/24/2024].

© 2024 Mahmoud H et al. *JAMA Network Open.*
