# Supplementary material for: Hospital Capacity Data and Extreme Heat Event Vulnerability
Source: JAMA Netw Open. 2024 Sep 11;7(9):e2432578. doi: 10.1001/jamanetworkopen.2024.32578 (PMC11391326; doi:10.1001/jamanetworkopen.2024.32578)
Supplement: Supplement 2. — Data Sharing Statement [file jamanetwopen-e2432578-s002.pdf]

Data Sharing Statement

Mahmoud. Hospital Capacity Data and Extreme Heat Event Vulnerability. *JAMA Netw Open*. Published September 11, 2024. doi:10.1001/jamanetworkopen.2024.32578

Data
Data available: Yes
Data types: Data (not involving human participants)
How to access data: hussam.mahmoud@colostate.edu
When available: With publication

Supporting Documents
Document types: Statistical/analytic code
How to access documents: hussam.mahmoud@colostate.edu
When available: With publication

Additional Information
Who can access the data: anyone requesting the data
Types of analyses: for any purpose
Mechanisms of data availability: with investigator support
